# Microbiological Quality of Foodstuffs Sold on Expiry Date at Retail in Portugal: A Preliminary Study

**DOI:** 10.3390/foods9070919

**Published:** 2020-07-13

**Authors:** Rita Maio, Juan García-Díez, Cristina Saraiva

**Affiliations:** 1 Department of Veterinary Sciences, School of Agrarian and Veterinary Sciences, University of Trás-os-Montes e Alto Douro, Quinta de Prados, 5001-801 Vila Real, Portugal; rita.hellokitty@hotmail.com (R.M.); crisarai@utad.pt (C.S.); 2 CECAV—Animal and Veterinary Research Centre, University of Trás-os-Montes e Alto Douro, Quinta de Prados, 5001-801 Vila Real, Portugal

**Keywords:** shelf life, foodborne, food waste, supermarket

## Abstract

Currently, food waste represents an important issue due to its negative economic, social and environmental impact. To reduce the food waste levels, some retailers’ brands implement discounting based on the proximity to expiry. Since this practice may involve potential food poisoning, a total of 94 food products from animal origin, purchased in two supermarkets in North Portugal on the expiry date, were analyzed for selected foodborne and spoilage microorganisms. Moreover, the samples were classified as satisfactory and not satisfactory according to their microbiological quality. The results showed that none of the samples presented counts for *Salmonella* spp., *S. aureus, B. cereus. L. monocytogenes* was detected in one sample over the limit of 2 log cfu/g as defined by Regulation 2073/2005. The evaluation of food hygiene and spoilage indicators showed that the processed foods displayed lower counts than raw products (beef, pork, chicken and fish). Regarding Enterobacteriaceae, raw products presented on average over 2 log cfu/g than processed foods, with the exception of beef samples that accounted over 3 log cfu/g more than processed foods. In addition, *E. coli* was mainly detected in fresh meat of which chicken and pork displayed the highest counts. Regarding the qualitative classification, 51.06% of the samples were not satisfactory for the total mesophilic counts, while 62.76% and 58.51% displayed positive results for Enterobacteriaceae and molds and yeasts (M&Y) criteria, respectively. In all, 70.21% of the samples analyzed at the expiry date failed, at least, in one microbiological criterion. The results indicate that the foods available at the end of the shelf life in supermarkets do not represent a risk for food poisoning due to the absence of foodborne pathogens. Since the microbiological indicators of storage/handling of raw products were mainly unsatisfactory, this indicates that the sale of these perishable foods at the end of the shelf life may not be recommended. On the other hand, processed products subjected to food conservation procedures (i.e., thermal processing) could be sold at the end of their shelf life or donated beyond the best-before date, due to its physical, chemical and microbiological stability. However, evidences of foodborne outbreaks associated to this kind of foodstuffs indicated the need of a proper risk assessment. Moreover, it is important to remark that other factors such as small sample size, the absence of the evaluation of the handling, and storage conditions along the food chain or organoleptic alterations must be assessed in further studies.

## 1. Introduction

In the European Union (EU), food labels have to mention the date of minimum durability or the ‘use by’ as defined by law [1]. The date of minimum durability should be expressed in day, month and year, in that order and in uncoded form. In addition, the date shall indicate the mentioned “best before” when the date includes an indication of the day or indicate the mention “Best before end” in other cases. However, food-labeling policy indicates some variations in the type of expression according to the food durability as well the absence of the compulsory mention of the date of minimum durability in specific foods. In the case of foods which, from a microbiological point of view, are highly perishable, the date of minimum durability shall be replaced by the ‘use by’ date. After the ‘use by’ date, a food shall be deemed to be unsafe as defined by law [2]. The differences among the “best before” and “used by” have created some misinterpretations among consumers between what is edible and what is not [3]. This is also supported by the lack of interest of consumers in the reading of the mentions displayed in food labels [4]. Thus, foods that have passed the “best before” date can be consumed if the storage conditions were properly achieved. In contrast, foods that have passed the “used by” date should not be consumed as defined by law as explained above. Food companies are responsible for determining the shelf-life of their foodstuffs and they always make sure to set the date at least several days before the product is no longer safe. Thus, each manufacturer determines its own margin of safety and ensures that the food product is consumed long before it becomes inedible or unsafe. In recent years, food sustainability has become a current concern, and is one of the main lines of the sustainable development goals of the United Nations [5]. Thus, the optimization of the expiry date of food products was considered one of the main points of action in view to ensure sustainable consumption and production patterns. This is because much of this food waste is due to the fact that large food retailers are forced to remove from the market huge amounts of foods that exceed both the “best before” and “use-by” dates [6] as well the unwillingness of supply chains and consumers to sell, purchase, and consume suboptimal or imperfect foods [7]. In the EU and based on the data from 2012 [8], it is estimated that about 88 million tons of food waste is generated annually with not only the negative economic and social impact, but also the environment depletion of limited natural resources.

Supermarkets generate less food waste than other food business operators (FBO) throughout the food chain [9]. However, the consumers’ preference at the retail level may contribute to food waste since they do not accept foods with sensory defects, even if that food could be sold in supermarkets [10].

At the retail level, bread, fruits, vegetables and pastry foods are the main categories of food waste accounting for about 30%, 14%, 13% and 12.5% respectively. Raw products such as beef, chicken or pork represent about 12% of food waste at the supermarket. Although the waste quantity is less than other categories, the food waste from raw products have the largest environmental impact [10]. Moreover, economic losses are not only associated to the lack of sale, but also derived from the cost of management by a by-product company. Thus, the optimization of stocks and the shelf-life of foods by retailers is essential to reduce the food waste.

To reduce the food waste levels in the retail sector, some retailer brands have implemented a discounting based on the proximity to expiry. Although the consumers´ choice of this kind of product is affected by social or economic factors, it has been described that budget saving or food waste avoidance does not seem to influence their choice of products [11]. In contrast, it seems that consumers have a perception of a lack of food safety in those products as they approach their end of shelf life [12,13].

The sale of foods close to or at the end of the expiry date may involve some health risks since the date of minimum durability may vary with some conditions, such as the inadequate handling of packages or variations in the storage temperature along the supply chain, among others. Thus, the objective of this work was to evaluate the microbiological hygiene and spoilage indicators of different food products sold in supermarkets at the expiry date in order to obtain knowledge about their safety and the perspective of food waste reduction. 

## 2. Material and Methods

### 2.1. Microbial Analysis

To evaluate the microbiological hygiene and spoilage indicators of foods on the expiry date, a preliminary study including 94 samples from animal origin were purchased in two supermarkets in North Portugal between 1 March 2019 and 31 May 2019. The basis of the selection was based only on the availability of foodstuffs at the supermarket at the end of the shelf life. The purchased foods were kept in their original packaging and transported in 30 min to the laboratory, in portable coolers and stored at 2 °C until the microbiological analysis was performed within 2 h. For the microbiological analysis, the foods products were aseptically opened and the samples were analyzed twice. Ten grams of the sample (25 g for *Salmonella* spp. and *L. monocytogenes*, respectively) were aseptically weighed, added to 90 mL of sterile peptone salt solution [14], and homogenized in a stomacher (Lab Blender, UK) for 60 s. The serial decimal dilutions in peptone salt solution were prepared and 1 or 0.1 mL of the appropriate dilutions were poured or spread on non-selective and selective agar plates, respectively.

Total mesophilic counts were enumerated in plate count agar media (PCA; Liofilchem, Teramo, Italy), incubated at 30 °C for 72 h and 7 °C for 10 days, respectively; lactic acid bacteria (LAB) in De Man, Rogosa and Sharpe agar (MRS; Liofilchem, Teramo, Italy) (30 °C, 72 h); Enterobacteriaceae (ENT) in violet red bile glucose agar (VRBG; Liofilchem, Teramo, Italy) (35 °C, 24 h); molds and yeasts (M&Y) on supplemented Rose-Bengal Chloramphenicol agar (Oxoid, Lenexa, KS, USA) (25 °C, 3–5 days); *Staphylococcus aureus* (SAU) on Baird Parker agar (Liofilchem, Teramo, Italy) supplemented with egg yolk tellurite (Difco, Sparks, MD, USA) and sulfamethazine (37 °C, 48 h); *Bacillus cereus* (BC) on Manitol Egg Polymyxin Agar (Liofilchem, Teramo, Italy) (30 °C, 24 h); and the *Escherichia coli* counts were obtained after incubation on Tryptone-bile-glucuronic medium (TBX) (Himedia, Mumbai, India) (41.5 °C, 24 h). *Pseudomonas* spp. (PSD) were determined using a Pseudomonas agar base (Oxoid) and Cefuxine, Fuxidin Cefradine (CFC) supplement (Oxoid, Lenexa, KS, USA) (30 °C, 48 h). The detection of *Salmonella* spp. (SAL) were performed as described elsewhere [15]. The enumeration of *Listeria monocytogenes* (LM) was obtained from 25 g of the sample using the University of Vermont medium (UVM)-I (20 °C for 1 h) and the Chromagar Listeria medium with Chromagar Identification Listeria and detection using UVM-I (30 °C for 24 h), UVM-II (37 °C for 24 h) and Oxford and Chromagar Listeria medium. The results are presented as log cfu/g. For statistical purposes, when the microorganism count was below the detection limit, it was considered to be zero log cfu/g. 

### 2.2. Data Analysis

According to the microbiological hygienic and spoilage results, the foods were classified as satisfactory and not satisfactory based on the criteria defined by food law. In these cases, in which no microbiological criteria were defined by law, the microbial criteria from the scientific reports were assumed. All the criteria for the different foodstuffs are presented in Table 1. In order to study the differences concerning the microbiological counts among the different foodstuffs, they were classified into two groups: raw products (beef, chicken, fish/fishery, meat products, pork) and processed products (cooked meal, pastry, ready-to-eat (RTE) dairy and RTE meat products). The foods included in each group were beef (steak, minced meat); chicken (whole and cut fresh chicken), fish and fishery (surimi, fresh sliced salmon, pilchard, Nile perch, fresh shrimp), meat products (traditional pork products such as *alheira—*pork sausage, *moura*—blood sausage, *farinheira*—mix of cornmeal and pork sausage), pastries (croissants, cream cake, chocolate cake, cheese cake, crap puffs), RTE dairy products (fresh cheese, yogurt, cottage cheese), and RTE meat products (cooked pork sausages, pork patties, cooked ham, cured chouriço, mortadella, meat cake). Statistical analyses were performed by one-way ANOVA. Comparisons of the means were obtained by the Tukey HSD test, for a significance level of *p* < 0.05. The quality classification of the samples according to the microbiological counts considered the worst result obtained in one of the spoilage/foodborne pathogens tested for each sample. All statistical analyses were completed using the SPSS Statistics Software (version 21, IBM, New York, USA).

## 3. Results

### 3.1. Microbiological Results of Food Groups

The microbiological evaluation of 94 food products purchased at the retail level on the expiry date (Table 2) showed that none of them presented counts for *Salmonella* spp., *S. aureus* and *B. cereus*. Only one traditional meat sausage displayed counts of *L. monocytogenes* over the limit of 2 log cfu/g as defined by law for this criteria. Regarding the spoilage and hygienic indicators, statistical differences (*p* < 0.001) among the raw products and processed products were observed for all of the microbiological groups with the exception of lactic acid bacteria. 

As expected, the evaluation of hygiene and spoilage indicators displayed the lowest microbiological counts in the processed products rather than the raw products. Regarding the total mesophilic counts, the raw products displayed similar counts (*p* > 0.05), between 5.93 and 6.17 log cfu/g while the processed foods presented lower counts (about 4.00 log cfu/g). However, it is important to remark that the highest counts observed for the meat products and dairy products are associated with the presence of cured or fermented products in these groups.

Regarding Enterobacteriaceae, raw products presented on average over 2 log cfu/g more than the processed foods, with the exception of beef samples, that accounted over 3 log cfu/g more than the processed foods. In addition, the *E. coli* counts ranged from 0.32 to 1.99 log cfu/ for the raw products while *E. coli* was not detected in the processed foods.

Spoilage indicators presented higher counts in the raw products than in the processed products. Thus, the LAB counts were about 5 log cfu/g in the raw products whilst 3.5 log cfu/g was observed in the processed products (without considering dairy products due to its fermented nature). Similar results were observed for Pseudomonads, which were higher in the raw products than in the processed products. Although the LAB presented large variations in the microbiological counts, no differences were observed (*p* > 0.05) within the food group. 

Pseudomonas counts ranged from 3.58 to 6.00 log cfu/g and 0.96 to 2.77 for the raw and processed foods, respectively. Th only differences (*p* < 0.05) in the microbiological counts were observed for the Pseudomonads in processed products.

### 3.2. Classification of Food Samples by Microbiological Criteria

The qualitative classification of food samples (Table 2) according to their microbiological results showed that 51.06% of samples were not satisfactory regarding the total mesophilic counts. In contrast, 62.76% and 58.51% displayed satisfactory results for Enterobacteriaceae and M&Y criteria, respectively. Regarding *E. coli* and *L. monocytogenes,* only two (2.12%) and one (1.06%) samples, respectively, were not satisfactory. In all, 70.21% of samples (67 out 94) analyzed on the expiry date failed, at least, in one microbiological criterion. Thus, according to the number of unsatisfactory criteria, 28.73% failed one criterion, 21.28% failed two criteria, 19.14% failed three criteria and 1.06% failed four criteria. 

The qualitative classification of the raw food samples according their microbiological results showed that 64.54% of samples were considered not satisfactory for the total mesophilic counts, 80.17% for Enterobacteriaceae and 57.61% for M&Y. Almost 100% of the samples were satisfactory with regards to *E. coli*. By group, over 50% of the beef samples were not satisfactory for the total plate count (TPC), Enterobacteriaceae and M&Y overall. Over 60% of the chicken samples were satisfactory for TPC and M&Y, although almost 90% were not satisfactory for Enterobacteriaceae. 

Regarding the fish, over 65% of the samples were not satisfactory for TPC, Enterobacteriaceae and M&Y. Similar results were observed for the meat products and pork in which 60% of samples were not satisfactory for TPC, Enterobacteriaceae and M&Y. 

When all the criteria were considered, 25 out 26 samples (96.15%) were considered as not satisfactory since they failed in at least one of the microbiological criteria (TPC, Enterobacteriaceae, M&Y and *E. coli*) studied.

Regarding the processed foods, 47.05%, 20.58% and 35.29% were considered not satisfactory for TPC, Enterobacteriaceae and M&Y, respectively. In contrast, all the samples displayed satisfactory results for *E. coli* as observed for the raw food samples. By group, 61.53%, 84.61% and 65.38% of the cooked meal samples were classified as satisfactory for the total mesophilic counts, Enterobacteriaceae and M&Y. The pastry showed negative results since over 60% of the samples analyzed were not satisfactory for the total mesophilic counts and M&Y. However, 60% of them were classified as satisfactory for Enterobacteriaceae. Over 55% of the RTE dairy foods were acceptable for Enterobacteriaceae and M&Y. With regards to the total mesophilic counts, almost 80% of them were expectedly not satisfactory, since they are fermented products as previously referred. RTE meat products displayed the best results since over 70% of samples were, at minimum, satisfactory.

## 4. Discussion

Food waste is a worldwide problem that has gained importance in recent years, both in the public and political agenda, especially by the need to feed a rising world population [8]. Current estimates indicate that approximately one third of the food produced worldwide for human consumption is wasted or lost, with the resulting economic and environmental cost [16]. The causes are not always the same and vary according to the type of foodstuffs, production, packaging, transport or storage. Moreover, the socio-economical characteristics of consumers such as sex, education or economical income among others contribute to this problem. In the European Union, it is estimated that about 88 million tons of food are wasted annually, with an estimated 5% (4.5 million tons) related to the retail sector [17]. In the retail sector, some food operators implemented a discounting based on the proximity to expiry in which the retailer reduces the price of foods according to its remaining shelf-life. This practice is developed not only to reduce the food waste, but also to avoid food waste management costs. The expiry date is indicated with a large safety margin, however some factors such as alterations along the cold chain or improper handling may compromise the food safety. Although this practice contributes to reduce food waste, it is also necessary to consider the potential impacts on the sensory characteristics, physical and chemical changes of foods along the storage period, consumers´ purchase habits and food safety. Research about foodstuffs sold on the expiry date is very scarce and mainly related to consumer behavior rather than food safety. However, it has recently reported that the combination of discounting practice with dynamic shelf life (using predictive microbiology) in fresh meat improved both the safety of the product and decrease the food waste [18]. 

Thus, the evaluation of the microbial quality of different food categories was based on the comparison with microbiological surveys at the retail level. In addition, microbial comparison for some food categories such as meat products, pastry or cooked meat is difficult due to the large variety of products, the absence of research on the expiry date and also the absence of specific microbiological criteria at the end of the shelf life. Indeed, to the authors´ best knowledge, this is the first report about the microbiological quality of foodstuffs sold in Portugal at the expiry date. 

In overall, the results showed that the risk of food poisoning is low since *Salmonella* spp., *E. coli*, *L. monocytogenes* and *S. aureus* were not detected. On the other hand, the evaluation of non-pathogenic organisms aims to evaluate the handling and storage practices. Since these microorganisms can reach food from different sources (i.e., food handlers, the environment), its study indicates the existence of favorable conditions for its growth. Most of the samples analyzed were classified as not satisfactory for TPC and Enterobacteriaceae, indicating that the storage and handling/processing conditions may not be adequate throughout the product shelf life.

With regards to pork meat at the retail level, the expected lower counts ranged from 3.5 to 4.5 log cfu/g have been reported [19,20] (3 log cfu/g less). *E. coli* counts were similar as described in the literature [20] however, the higher counts about 2.5 log cfu/g (two-fold more than our study) at the retail level have been reported [19]. The microbiological condition of beef at the end of the shelf life presented some differences as reported in other works during the storage period [21,22]. Thus, the counts for TPC and LAB in our study displayed higher counts of about 1 log cfu/g, 2 log cfu/g and 2 log cfu/g for TPC, LAB and ENT. In contrast, similar results were observed for *Pseudomonas* spp. and *E. coli* [22]. Microbiological quality surveys of chicken at the retail level showed similar results to our study at the expiry date. Thus, the total mesophilic counts differ at about 0.5 log cfu/g [23,24]. Regarding hygienic indicators, our study presented, on average, less than 2 log cfu/g for *E. coli* [24,25,26]. Spoilage indicators (*Pseudomonas* spp. and M&Y) presented similar to those observed by other authors [24,27]. Results may indicate proper storage since the spoilage values related to *Pseudomonas* spp. are associated to counts about 7–8 log cfu/g. M&Y are not part of the natural microflora of chicken [28]. However, its relationship with the shelf life may explain the similar counts of the other spoilage indicators studied [29]. The presence of the foodborne pathogens such as *Salmonella* spp., *L. monocytogenes* or *S. aureus* at the retail level for fresh meat (pork and beef) and chicken has been previously reported, although with variable prevalence [20,30,31] mainly related to differences in the sanitation of retail butcher shops and the hygienic standards of food handlers. Regarding *Salmonella* spp., the most common cause of the human foodborne pathogen linked to poultry, prevalence values at the retail level ranged from 30% to 50% [27,32], although recent reports showed lower prevalence [28], indicating that the poultry industry apparently undertook effective measures for its control [33]. Despite the small sample size, foodborne pathogens were not detected. However, it is important to remark that the potential presence of these pathogens can reach infectious levels (at the expiry date), especially if storage or inadequate handling conditions throughout the shelf life have not been respected. 

Fish is a highly perishable food and with a variable microbiota, ranging from 2 to 7 log cfu/g and influenced by factors such as the local fish species or packaging system. During the chilling storage, *Pseudomonas* and *Alteromonas* are the predominant genus. 

The study of the microbial quality of fresh fish carried out by Van den Broek [34] showed that about 20% and 60% of the samples analyzed displayed TPC counts of about 5–6 log cfu/g and 6–7 log cfu/g, respectively, in accordance with our results. However, only 43% of the fish samples displayed similar values for ENT in accordance with our results. In contrast, other authors [35] reported ENT counts over 1 log cfu/g as well as the absence of *E. coli* in accordance with our results. Since *Salmonella* spp. infection transmitted by fish is uncommon and *S. aureus* is not a natural inhabitant of seawater fish, the absence of them in our samples may be explained. However, our results regarding the foodborne pathogens must be carefully interpreted since pathogens such as *L monocytogenes* or *Vibrio* spp. have been described in fresh fish at the retail level with special relevance when destined to be consumed raw [35,36], undercooked or very lightly processed [37,38].

Other reports [39] on RTE meat products at the end of shelf life, showed similar results for the total mesophilic counts, M&Y, *E. coli* and *Salmonella* spp., although the authors also reported the presence of *L. monocytogenes* and *S. aureus* in levels under the legal limit of 2 log cfu/g defined by law for *L. monocytogenes* [40] and unable for the toxin production of *S. aureus* [41]. Thus, our results seem to guarantee the safety of these products even at the end of the shelf life.

In the case of the sliced RTE meat products, Perez-Rodriguez [42] reported similar results for non-pathogenic bacteria. In addition, the low levels of ENT and *E. coli* observed in our samples suggest an adequate hygiene of processing/handling. Since the slicing process is recognized as a risk procedure regarding foodborne contamination, the consumption of sliced meat products at the end of the shelf life, even with the absence of foodborne pathogens in studied samples, may be appropriately evaluated [43]. 

The microbiological evaluation of cooked meals and pastry foods is very difficult due to the large variety of foods, ingredients used and regional characteristics. Cooked meals represented the main foods sold at the retail level on the expiry date, probably associated to their short shelf life. The absence of foodborne pathogens in cooked meals is predictable due to its thermal processing (i.e., cooking). Regarding non-pathogenic microorganisms, the results showed that most of them were considered at least satisfactory. Thus, microbiological counts for total mesophilic counts, ENT, LAB and M&Y were expectedly higher than reported [44]. However, another study [45] showed lower total mesophilic counts for based-meat meals after 30 days of cold storage. Moreover, low ENT counts and the absence of foodborne pathogens have also been reported [46]. 

Although the safety of cooked meals seems to be guaranteed, the fact that about 40% of cooked meals fail the total mesophilic counts criteria suggests some deficiencies along the cold storage and/or food handling. 

The safety of pastry products is guaranteed by its constitution (low a_w_) and manufacture (oven baked). The total mesophilic counts ranged from 2 to 4 log cfu/g at the retail level [47], however, cream-filled pastry products seem to have higher counts for the total mesophilic counts, Enterobacteriaceae and M&Y [48,49]. Higher counts for the total mesophilic and Enterobacteriaceae obtained in pastry products than those observed in our samples have been reported [47]. According to the microbiological criteria, our samples displayed a higher percentage of non-satisfactory levels, contrarily to what was described [50]. However, better performance regarding hygienic indicators and foodborne pathogens were obtained regarding the presence of *B. cereus*, *Salmonella* spp. and *L. monocytogenes* [47]. Even though neither *L. monocytogenes* nor *Salmonella spp*. were detected in the present study, this report indicates that these kinds of products support the growth of foodborne pathogens. In consequence, the food handlers’ hygiene during handling and preparation is necessary to avoid cross-contamination.

Among dairy products, yogurts, fresh and cottage cheeses are the principal foods sold at the end of their shelf life in supermarkets. The high counts for the total mesophilic and LAB are expected as they are fermented products [51]. In the case of yogurts, their low pH acts as a barrier for pathogenic bacteria growth. In the case of fresh or cottage cheeses, the absence of foodborne pathogens may be explained due to the thermal processing (i.e., pasteurization) and vacuum packaging. However, the presence of pathogenic microorganisms in retail cheese made from pasteurized milk have been reported [52] indicating that these products support its growth. 

Nowadays, based on the experience of economic crisis in recent years, the practice of food donation to social institutions has gained great importance. Due to the potential impact on public health, the European Food Safety Authority (EFSA) released a scientific opinion about the risks of this practice [53]. The present work showed that most of the raw products were classified as not satisfactory for the storage and handling of microbial indicators. Although foodborne pathogens were not identified, potential changes in the organoleptic properties, mainly derived from spoilage, suggest that these products should not be sold at the end of the shelf life. 

On the other hand, the lower microbiological counts observed in processed products may be related to their manufacturing process (i.e., heat treatment, fermentation or ripening). Given their stability, these foods could be considered as appropriate for donation beyond the best-before date. In addition, an increase in the expiry period by food manufacturers as a measure to reduce food waste have been proposed [54] but, according to our results, it may only be suggested for processed products. However, this practice must be carefully assessed since some of these products (as evidenced in one meat product), despite its stability, have demonstrated their ability to carry foodborne pathogens.

## 5. Conclusions

The results of the 94 food samples analyzed in the present study indicate that the foods available with discount based on proximity to the end of the shelf-life, at supermarkets, do not represent a risk for food poisoning due to the absence of foodborne pathogens. Since the microbiological indicators of the storage/handling of raw products were mainly unsatisfactory, the sale of these raw foods at the end of their shelf life may not be recommended. In addition, the data obtained highlights the need to improve proper hygienic and storage practices during the products shelf life. On the other hand, processed products could be donated beyond their best-before date due to their stability based on their manufacturing process. However, evidences of foodborne outbreaks associated to this kind of foodstuffs indicated the need of a proper risk assessment. 

Moreover, it is important to remark some limitations of the present study, such as the small sample size, the absence of compliance with the food sampling requirements as defined by law, the absence of the evaluation of the handling or storage conditions along the food chain, among others, which can influence the microbial characteristics at the end of the shelf life. Although the work accounts for the issues of microbiological spoilage, the authors are also aware that other abiotic concerns (i.e., dehydration, discoloration, enzyme activity) may affect the foods at the expiry date. Thus, all factors previously described must be assessed in further studies. In addition, the large variety of foodstuffs and the lack of specific microbial criteria is a challenge for its comparison.

## Figures and Tables

**Table 1:** Microbiological criteria (expressed in log cfu/g) for foodborne and spoilage bacteria in different foods groups.

Food	Class.	TMC	MY	ENT	ECO	LIS	BAC	SAL	SAU
Meat	Sat	≤6	≤4	≤2.5	≤3	<2	≤5	Abs. in 25g	<2
	NSat	>6	>4	>2.5	>3	≥2	>5	Pres. in 25g	≥2
	Ref	a	c	a	a	a	e	a	g
Dairy	Sat	≤4	≤3	≤3	≤3	<2	≤5	Abs. in 25g	≤3
	NSat	>4	>3	>3	>3	≥2	>5	Pres. in 25g	>3
	Ref	b	b	b	a	a	e	a	a
Pastry	Sat	≤3	≤2	≤2	<1	Abs. in 25g	≤5	Abs. in 25g	<1
	NSat	>3	>2	>2	≥1	Pres. in 25g	>5	Pres. in 25g	≥1
	Ref	b	d	a	b	a	e	a	b
Fish	Sat	≤5	≤4	≤3	Abs.	Abs. in 25g	≤5	Abs. in 25g	Abs. in 0.01g
	NSat	>5	>4	>3	Pres.	Pres. in 25g	>5	Pres. in 25g	Pres. In 0.01g
	Ref	f	c	h	f	f	e	f	f
Cooked meals	Sat	≤4	≤3	≤2	<1	<2	≤5	Abs. in 25g	≤4
	NSat	>4	>3	>2	≥1	≥2	>5	Pres. in 25g	>4
	Ref	b	b	a	b	a	e	a	b

Class: classification; TMC: total mesophilic counts; MY: moulds and yeast; ENT: Enterobacteriaceae; LAB: lactic acid bacteria; PSE: Pseudomonads; ECO: *E. coli*; LIS: *L. monocytogenes*; BAC: *Bacillus cereus*; SAL: *Salmonella* spp.; SAU: *S. aureus*; SAT: satisfactory; NSat: not satisfactory; Ref: reference; nd: not defined; Pres: presence; Not pres.: not presence; Abs: absence; pres: presence; meat criteria includes beef, chicken, pork and meat products. a: Regulation (EC) No 1441/2007 of 5 December 2007 amending Regulation (EC) No 2073/2005 on microbiological criteria for foodstuffs. http://data.europa.eu/eli/reg/2007/1441/oj. b: Santos MI, Correia C, Cunha MIC, Saraiva MM, Novais MR (2005) Valores Guia para avaliação da qualidade microbiológica de alimentos prontos a comer preparados em estabelecimentos de restauração. Revista da Ordem dos Farmacêuticos 64:66–68. c: Fung, D. Y. C. (2014) Yeast and molds. In: Encycplopedia of meat sciences. 2° Ed. Dikeman, M., Devine, C. (Eds). Academic Press. London, UK. pp. 395–404. d: Real Decreto 135/2010, de 12 de febrero, por el que se derogan disposiciones relativas a los criterios microbiológicos de los productos alimenticios. https://www.boe.es/boe/dias/2010/02/25/pdfs/BOE-A-2010-3032.pdf. e: European Food Safety Authority (EFSA). (2005). Opinion of the Scientific Panel on biological hazards (BIOHAZ) on *Bacillus cereus* and other *Bacillus* spp. in foodstuffs. *EFSA Journal*, *3*(4), 175 f: Official control of fish and fishery products of exporting establishments. Spanish Government. 2013. https://www.mscbs.gob.es/profesionales/saludPublica/sanidadExterior/docs/pescaUA.pdf. g: Orden de 14 de enero de 1986 por la que se aprueba la norma de calidad para carnes picadas de vacuno, ovino y porcino destinadas al mercado interior. https://www.boe.es/eli/es/o/1986/01/14/(2).

**Table 2:** Microbial counts expressed as log CFU/g (mean ± standard deviation) and microbiological quality classification of different foodstuffs according to its type.

	Food Type	Micro Criteria	Microorganisms					
			Total Plate Count	Enterobacteriaceae	Lactic Acid Bacteria	Moulds and Yeast	Pseudomonads	*E. coli*
Raw products (RP)	Beef		6.17 ± 1.56	4.18 ± 1.36	4.91 ± 0.84 ^ab^	3.79 ± 1.22	3.02 ± 3.34	0.32 ± 0.65
		Sat	2	0	-	1	-	4
		NSat	2	4	-	3	-	0
	Chicken		5.94 ± 1.21	3.74 ± 1.47	3.58 ± 0.59 ^b^	3.42 ± 1.63	4.82 ± 2.01	1.09 ± 1.52
		Sat	5	1	-	5	-	5
		NSat	2	6	-	2	-	2
	Fish/fishery		5.71 ± 2.94	2.74 ± 2.22	4.05 ± 0.69 ^ab^	3.45 ± 1.95	5.55 ± 2.93	nd
		Sat	1	2	-	2	-	6
		NSat	5	4	-	4	-	0
	Meat products		7.19 ± 1.52	3.27 ± 3.39	6.90 ± 0.75 ^a^	2.76 ± 2.67	2.63 ± 2.19	nd
		Sat	1	2	-	3	-	5
		NSat	4	3	-	2	-	0
	Pork		6.72 ± 0.89	2.91 ± 2.88	5.32 ± 0.97 ^ab^	5.07 ± 0.62	3.81 ± 3.79	0.99 ± 1.72
		Sat	1	0	-	0	-	3
		NSat	2	3	-	3	-	0
	*p*		ns	ns	*p* < 0.01	ns	ns	ns
Processed products (PP)	Cooked meal		3.98 ± 2.01 ^b^	0.50 ± 1.14	2.97 ± 2.14 ^b^	1.69 ± 1.82	2.77 ± 2.09 ^a^	nd
		Sat	16	22	-	17	-	27
		NSat	11	5	-	10	-	0
	Pastry		4.39 ± 2.04 ^ab^	1.16 ± 1.54	2.84 ± 2.32 ^b^	2.12 ± 1.90	2.36 ± 2.29 ^ab^	nd
		Sat	1	5	-	3	-	9
		NSat	8	4	-	6	-	0
	RTE dairy		6.83 ± 3.35 ^a^	1.37 ± 2.24	6.44 ± 2.99 ^a^	2.21 ± 2.25	2.30 ± 2.54 ^ab^	nd
		Sat	2	7	-	5	-	9
		NSat	7	2	-	4	-	0
	RTE meat products		4.70 ± 2.53 ^ab^	1.02 ± 1.40	3.92 ± 2.76 ^ab^	1.80 ± 2.10	0.96 ± 1.53 ^b^	0.04 ± 0.20
		Sat	17	20	-	19	-	24
		NSat	7	4	-	5	-	0
	*p*		*p* < 0.05	ns	*p* < 0.05	ns	*p* < 0.05	ns
*p (RP x PP)*			*p* < 0.001	*p* < 0.001	ns	*p* < 0.001	*p* < 0.001	*p* < 0.001

MO: microorganisms, Sat: satisfactory, NSat: not satisfactory, RTE: ready-to-eat, ns: not significant, Micro. Criteria: microbiological classification based on policy or scientific literature, Lactic acid bacteria and Pseudomonads not presented microbiological classification due to the absence of microbial criteria available at policy and/or scientific literature. Values in the same row with different superscripts letters are significantly different (*p* < 0.05).
